# Improved Biosafety and Transdermal Delivery of Aconitine via Diethylene Glycol Monoethyl Ether-Mediated Microemulsion Assisted with Microneedles

**DOI:** 10.3390/pharmaceutics12020163

**Published:** 2020-02-17

**Authors:** Yongtai Zhang, Hongmei Hu, Qian Jing, Zhi Wang, Zehui He, Tong Wu, Nian-Ping Feng

**Affiliations:** Department of Pharmaceutical Sciences, Shanghai University of Traditional Chinese Medicine, Shanghai 201203, Chinawangzhi908@163.com (Z.W.); 18817773761@163.com (Z.H.);

**Keywords:** nanomedicine, nanoemulsion, permeability, topical administration, pharmacokinetics

## Abstract

In the current study, diethylene glycol monoethyl ether-mediated microemulsions were combined with microneedles for enhanced transdermal aconitine delivery. The oil-in-water microemulsion increasedaconitine solubility and enhanced transdermal drug delivery and assistance with metal microneedles enhanced permeation of the aconitine-loaded microemulsion. Carried by the microemulsion, the in vitro permeability of aconitine was significantly enhanced, and further improved using microneedles. In vivo microdialysis revealed that the subcutaneous local drug concentration reached a high level within 30 min and remained relatively consistent to the end of the experimental period. AUC_0-t_ of the microemulsion group was significantly higher than that of the aqueous solution group, and the microemulsion combined with microneedles group achieved the highest AUC_0-t_ among the tested groups. The microemulsion and microdialysis probe also showed good biocompatibility with skin tissue. The microemulsion could be internalized by HaCaT and CCC-ESF-1 cells via lysosomes. The in vitro cytotoxicity of aconitine toward skin cells was reduced via encapsulation by microemulsion, and the prepared microemulsion developed no skin irritation. Hence, transdermal aconitine delivery and drug biosafety were effectively improved by loading into the microemulsion and assisting with microneedles, and in vivo microdialysis technique is suitable for realtime monitoring of transdermal drug delivery with microemulsion-based drug vehicles.

## 1. Introduction

**Figure 1:**
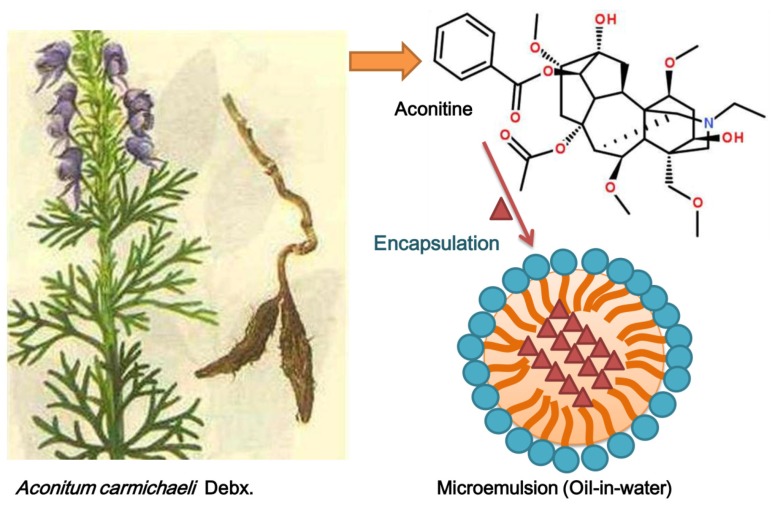
Chemical structure of aconitine and schematic diagram of the drug molecules being encapsulated into the microemulsion.

Aconitine is an active ingredient isolated from the herb *Aconitum carmichaeli* Debx., which is commonly used as an analgesic in traditional Chinese medicine, and transdermal administration can improve its safety [1]. Microemulsions, nanovehicles for transdermal drug delivery, involve isotropic, transparent, and thermodynamically stable dispersion systems spontaneously formed by the interaction between water, oil, surfactant, and co-surfactant in appropriate proportions [2]. Microemulsions have low surface tension, and they easily moisturize the skin and disrupt the firm structure of the stratum corneum [3]. In addition, they increase drug solubility, which can enhance drug permeation into the skin [4]. Aconitine-loaded microemulsions (Figure 1) have been demonstrated to improve transdermal drug absorption via topical administration [5].

However, because nanocarriers are disturbed by the stratum corneum barrier when penetrating the skin, it is difficult to control the amount of drug absorbed into the skin accurately, which is still a challenge for the safe use of drugs with narrow therapeutic windows. Microneedles are a new transdermal drug delivery technology developed in recent years [6]. When microneedles are used for transdermal drug delivery, the micro-sized needles can painlessly create microchannels in the skin, allowing the drug to directly enter the skin, thereby achieving precise administration [7]. The combination of microneedles and nanocarriers for transdermal administration can increase the solubility of drugs and the drug loading of microneedles, improve the stability and biosafety of drugs with nanocarriers, and achieve targeted drug delivery [8,9]. Therefore, the combination of a microemulsion and microneedles for transdermal delivery of aconitine can simultaneously increase drug solubility, enhance percutaneous absorption, and accurately administer drugs, thereby improving safe medication [10,11].

In order to accurately evaluate transdermal drug delivery using microneedles combined with nanotechnology, local microdialysis is a reliable tool. Microdialysis is a minimally invasive sampling technique that combines dialysis and continuous perfusion microsampling in vivo. The microdialysis probe is inserted into the tissue and perfused under non-equilibrium conditions, and the free, unbound analyte in the extracellular fluid diffuses through the membrane of the probe into the perfusate by the concentration gradient [12]. The advantage of microdialysis is that it can be used in vivo, in real time, and for online sampling without substantially interfering with normal life activities. Furthermore, it is particularly useful for studying dynamic changes in biochemical functions or determining the distribution of exogenous compounds in the body [13]. Microdialysis techniques have been widely used in the evaluation of dermal and transdermal drug delivery to enable the determination of drug distribution in the skin or subcutaneous tissue using in vivo sampling [14,15]. This technique has been used to successfully evaluate the transdermal absorption of various pharmaceutical ingredients loaded in nanosized carriers for topical administration [16,17].

In this study, aconitine was encapsulated into a microemulsion and combined with microneedles for transdermal administration. This enhanced transdermal aconitine delivery was evaluated in vitro, and the drug concentration in local tissue was monitored realtimeby in vivo microdialysis. In addition, the biocompatibility of the microdialysis probe in skin tissue and the cytotoxicity of the aconitine-loaded microemulsion toward skin cells were investigated.

## 2. Materials and Methods

### 2.1. Materials

Diethylene glycol single ethyl ether (Transcotol^®^ P) was obtained from Gattefossé (Lyon, France). Polyethylene glycol (PEG)-35 castor oil (Cremophor^®^ EL) was obtained from BASF (Ludwigshafen, Germany). Aconitine (purity> 98.0%) was supplied by Ze-lang BioScience (Nanjing, China). Methyl thiazolyltetrazolium (MTT) and materials used in cell culture were purchased from Gibco (ThermoFisher Scientific Inc., Grand Island, NY, USA). Hoechst 33,342 and LysoTracker Red were supplied by Santa Cruz Biotechnology, Inc. (Dallas, TX, USA). All other chemicals were purchased from the Sinopharm Chemical Reagent Co., Ltd. (Shanghai, China). 

Male Sprague–Dawley rats, weighing 300 ± 20 g, were provided by the Experimental Animal Center of Shanghai University of Traditional Chinese Medicine. The study protocol was approved in April 2019 by the Experimental Animal Ethics Committee of Shanghai University of Traditional Chinese Medicine (license numbers: SYXK [Hu] 2008-0050, SYXK [Hu] 2009-0069).

The HaCaT cell line was obtained from ATCC (Manassas, VA, USA), and the CCC-ESF-1 cell line was provided by the Cell Culture Center of the Chinese Academy of Medical Sciences (Beijing, China).

### 2.2. Detection of Aconitine and its Solubility

The aconitine assay was performed according to the protocol outlined in our previous report [5]. Briefly, the samples were filtered through a microporous filter (pore diameter: 0.45 μm), and then assayed by HPLC (LC-2010A, Shimadzu Corporation, Kyoto, Japan) with a chromatographic column (C18, ODS, 5 μm, 4.6 mm × 25 cm). The mobile phase consisted of methanol:water:trichloromethane:triethylamine (70:30:20:0.1, *v*/*v*/*v*/*v*) with a flow rate of 1 mL/min, and the eluate was monitored at 235 nm. Sample detection was conducted in triplicate.

Drug solubility was assessed by adding excess aconitine in pure water with the prepared microemulsion. The mixture was stirred at 25 °C for 72 h, centrifuged at 3000 × *g* for 10 min to remove sediment from the solution, and then assayed by HPLC.

### 2.3. Preparation and Characterization of Aconitine-Loaded Microemulsion

The lipid excipients, including ethyl oleate (8%, *w*/*v*) as the oil phase, cremophor^®^ EL (8%, *w*/*v*) as the emulsifier, and Transcotol^®^ P (24%, *w*/*v*) as the co-emulsifier, were mixed with the drug (0.25‰, *w*/*v*) and dissolved by stirring. Then, PBS (pH 7.4) (60%, *w*/*v*) was added dropwise to form a transparent and homogeneous microemulsion. Coumarin 6 was used instead of aconitine to prepare a coumarin 6-labeled microemulsionat 10 μg/mL for the study of cell uptake.

The aconitine aqueous solution (aconitine solution) was prepared by directly dispersing aconitine powder in PBS to a final drug concentration of 0.25‰ (*w*/*v*). The aconitine DMSO solution used for in vitro release and cell viability studies was prepared by dissolving the drug in DMSO and then diluting with PBS to obtain final concentrations of aconitine and DMSO of 0.25‰ (*w*/*v*) and 1% (*v*/*v*), respectively.

The size distribution and zeta potential of the aconitine-loaded microemulsion were respectively measured by a particle size analyzer (Particle Sizing SystemsInc. Santa Barbara, CA, USA) and a Zetasizer Nano ZS 90 Instrument (Malvern Panalytical, Malvern, UK) at 25 °C. The appearance of the microemulsion droplets was observed by transmission electron microscopy (TEM; Tecnai 12; Philips, Amsterdam, Netherlands).

The unencapsulated drug molecules were separated in an ultrafiltration tube (MWCO: 10 kDa) by centrifugation at 3000× *g*, and then assayed by HPLC. The entrapment efficiency and drug loading were respectively calculated according to the following Equations (1) and (2):

(1) $$\mathrm{entrapment}\;\mathrm{efficiency}\;\left(\%\right)=\frac{W_{t}-W_{f}}{W_{t}}\times 100$$

(2) $$\mathrm{drug}\;\mathrm{loading}\;\left(\%\right)=\frac{W_{t}-W_{f}}{W_{m}}\times 100$$

where W_t_ is the total weight of aconitine in the microemulsion system, W_f_ is the weight of free unencapsulated drug in the microemulsion, and Wm is total weight of lipid excipients and the drug in the microemulsion formulation. All of the above measurements were performed in triplicate.

### 2.4. In Vitro Release

One milliliter of microemulsion and the compared formulations were individually encapsulated in a dialysis bag (MWCO: 10 kDa). Tween 80 aqueous solution (1%, *w*/*v*) was used as the receiving medium for maintaining sink conditions. The in vitro release studies were performed at 37 °C. At a predetermined time point, a fixed volume of the receiving medium was sampled, and the remaining dialysate was replenished with an equal volume of fresh receiving medium preheated to 37 °C. The resulting sample was assayed by HPLC. Each test was performed in triplicate.

### 2.5. In Vitro Transdermal Evaluation

Each rat was anesthetized with urethane, the hair on the abdomen was removed with a razor, and the rat was euthanized. The abdominal skin was removed and washed with normal saline. Skin permeation studies were performed using a Franz diffusion cell; the stratum corneum of the rat skin faced the donor cell and the dermis faced the receptor cell. One milliliter of the tested preparation was added to the donor cell, and 16 mL of 20% PEG 400 solution (*v*/*v*) was added to the receptor cell, and the apparatus was maintained at 32 °C. For administration of microemulsion combined with microneedles, the rat skin was preprocessed by the metal microneedles (Medical-grade stainless steel needles; 600 needles, 0.25 mm; Derma Roller System Inc., Markham, Ontario) by rolling for 10 s, and the drug was then administered. Samples were removed from the receptor cell at predetermined times and assayed by HPLC.

### 2.6. In Vivo Microdialysis Experiments

The in vivo microdialysis experiments were conducted using Sprague–Dawley rats. The microdialysis probe (Spectrum Lab, Inc., Chicago, IL, USA) had a molecular weight of 13 kDa, an outer diameter of 280 μm, and an inner diameter of 200 μm, and the subcutaneous length of this probe was maintained at 2 cm. The diameter of the donor cell situated just above the probe was 1.5 cm, and this cell was filled with 1 mL of the drug-loaded microemulsion. The reference preparation was an aconitine aqueous solution with the same drug concentration as the tested microemulsion. Further, 20% PEG400 was the perfusate, and the flow rate was 3.33 μL/min. The subcutaneous local aconitine concentration was calibrated according to the recovery of the in vivo microdialysis that was obtained by reverse dialysis in a previous study [18]. Finally, local pharmacokinetic parameters were determined using a non-compartment model.

### 2.7. Histopathology of Skin Sections

After the in vivo microdialysis experiment was completed, the skin at the administration site was removed, fixed with 4% paraformaldehyde, and then embedded in paraffin and sectioned. The skin sections were stained with hematoxylin and eosin (H&E), and the pathological changes in the tissues were observed under a microscope (IX70; Olympus Corporation, Hatagaya, Japan).

### 2.8. Cell Viability

Cells at 5 × 10^3^ cells/well were incubated in the mixed solution of each tested preparation (aconitine solution, PBS containing 0.25‰ (*w*/*v*) aconitine and 1% (*v*/*v*) DMSO; aconitine-loaded microemulsion, containing 0.25‰ (*w*/*v*) aconitine; or empty microemulsion, microemulsion without aconitine) and DMEM (containing 10% FBS) in different volume ratios for 24 h. The medium was then replaced with fresh DMEM (without FBS) containing 500 μg/mL MTT, and the incubation continued for 4 h. The culture medium was removed, the generated formazan was dissolved in 200 μL of DMSO, and the optical density (OD) was assayed at 570 nm with a microplate reader (Synergy H1; BioTek Instruments, Inc., Winooski, VT, USA). Cell viability was determined by using Equation (3):

(3) $$\mathrm{Cell}\;\mathrm{viability}\;\left(\%\right)=\frac{{\mathrm{OD}}_{\mathrm{sample}}-{\mathrm{OD}}_{\mathrm{blank}\;\mathrm{control}}}{{\mathrm{OD}}_{\mathrm{normal}\;\mathrm{control}}-{\mathrm{OD}}_{\mathrm{blank}\;\mathrm{control}}}\times 100$$

### 2.9. Cell Uptake

Cells were incubated in the mixed solution of coumarin 6-labeled microemulsion and DMEM (10% FBS) at a volume ratio of 1:999 for 0.5 h, washed with PBS, and then cultured with fresh DMEM (without FBS) containing 50 nM LysoTracker Red for 30 min.Thereafter, Hoechst 33342 was added to a final concentration of 10 μg/mL and the incubation was continued for 5 min. The cells were fixed in 4% (*w*/*v*) paraformaldehyde for 15 min and observed under a laser confocal microscope (TCS SP5; Leica Microsystems, Wetzlar, Germany).

### 2.10. Preliminary Skin Irritation Test

The hair on the backs of the rats was shaved, before randomly dividing them into the normal skin group and the injured skin (skin was injured by drawing “#” on the skin with a blade, until capillary hemorrhage was observed) group. The hair on the back of each rat was shaved, and the test preparation was applied at 0.5 mL/cm^2^ once daily for 7 consecutive days. The rats were euthanized, and the skin at the administration site was removed, sectioned, and H&E stained according to the method in Section 2.7. The changes in the skin tissue were observed under a microscope.

### 2.11. Data Analysis

The data generated in the experiments are expressed as means ± SD. Statistical analysis between the two groups was performed using the *Student’s t*-test. A *p*-value less than 0.05 was considered a significant difference.

## 3. Results

### 3.1. Characteristics of the Prepared Microemulsion

**Figure 2:**
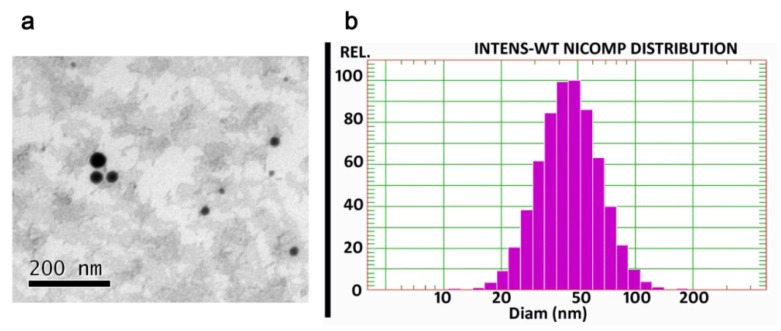
Transmission electron microscopy (**a**) and size distribution (**b**) of the aconitine-loaded microemulsion.

The microemulsion appeared as spheroidal droplets under TEM (Figure 2a). The size distribution of this colloidal solution was less than 100 nm (Figure 2b) and the zeta potential was −6.82 ± 0.48 mV. The entrapment efficiency of the drug in the microemulsion was 92.95 ± 11.02%. Due to the very low drug content in the formulation, drug loading of the microemulsion was 0.06 ± 0.01%.

Notably, the solubility of aconitine in water at 25 °C was only 12.25 ± 1.54 μg/mL, but it sharply increased to 3744.41 ± 254.36 μg/mL in the prepared microemulsion.

The drug was released rapidly from the aconitine DMSO solution (containing 1% (*v*/*v*) DMSO) within 0.5 h. The microemulsion showed a sustained release of the drug in contrast with that of the aconitine DMSO solution, with a cumulative release percentage of less than 50% within 5 h and more than 90% by 8 h. However, drug release from the aconitine solution (aconitine powder directly dispersed in PBS) was slower than that from the microemulsion group, with only <25% released after 24 h. This is mainly due to the strong solubilization of aconitine by the microemulsion.

In addition, when stored at room temperature (20–30 °C) for 1 week, the microemulsion size distribution and the drug entrapment efficiency did not change significantly (*p* > 0.05).

### 3.2. In Vitro Skin Permeation

**Figure 3:**
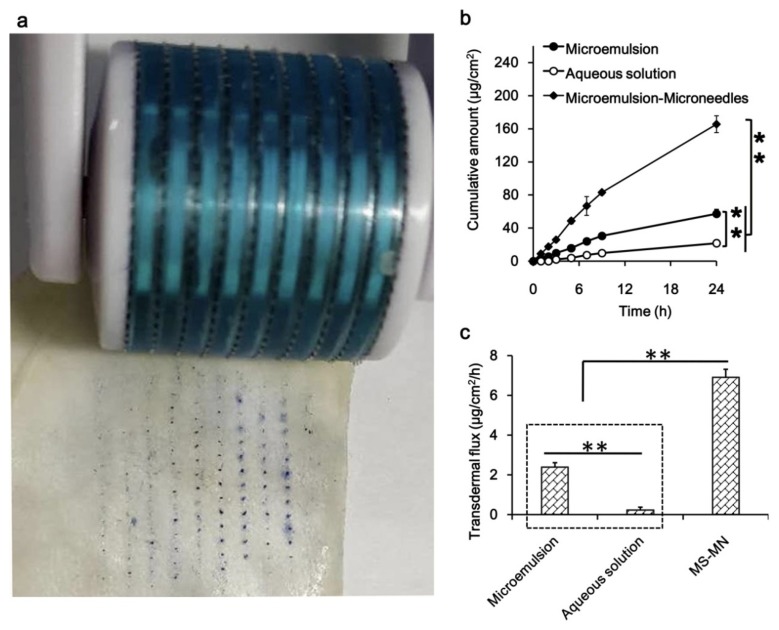
Microneedles and microneedle-treated rat skin (**a**), in vitro transdermal profiles (**b**), transdermal flux and skin deposition (**c**) of aconitine from microemulsion and the aqueous solution. ** *p* < 0.01, (*n* = 3).

The microneedle-treated skin surface clearly showed microchannels (blue pinholes stained with methylene blue), which become channels for the drug to be transported into the skin (Figure 3a). By loading into the microemulsion, the cumulative amount of drug permeated across the skin greatly increased in contrast with that of the drug in aqueous solution (Figure 3b), and the permeability of the drug was further enhanced by using microneedles. Correspondingly, the in vitro aconitine transdermal flux of the microemulsion group was significantly higher than that of the aqueous solution, and the microemulsion combined with microneedles group achieved the highest transdermal flux of aconitine (*p* < 0.01; Figure 3c). Notably, in view of the high toxicity of aconitine, it is recommended that the daily dose of the microemulsion preparation should be controlled within 1 mL.

### 3.3. In Vivo Microdialysis

**Figure 4:**
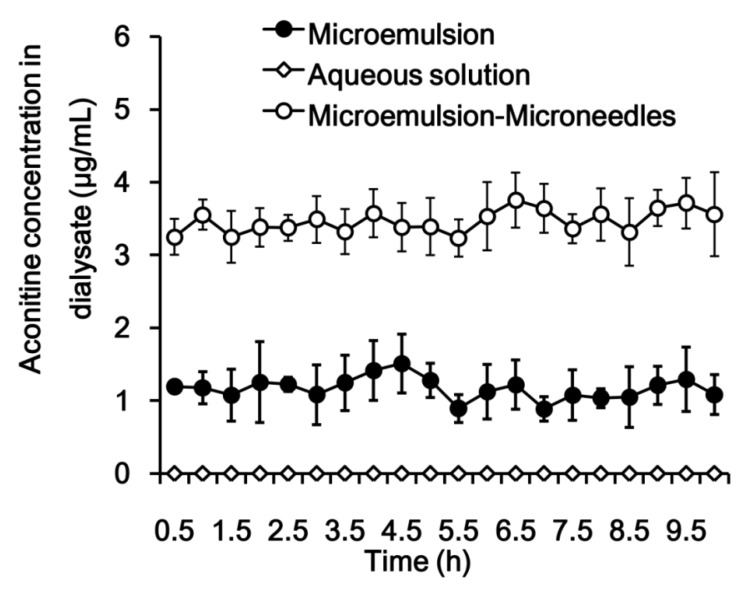
Aconitine concentration in deep skin tissue by using microdialysis sampling after application of aconitine-loaded microemulsion and aqueous solution to the abdominal skin of Sprague–Dawley rats in vivo (*n* = 3).

**Figure 5:**
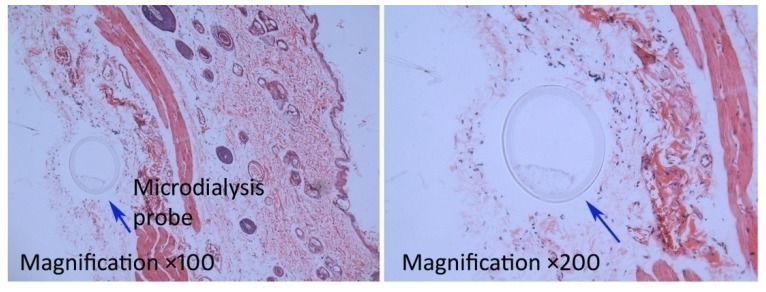
Micrographs of rat skin sections embedded in paraffin and stained with hematoxylin and eosin by using microdialysis sampling for 10 h after application of aconitine-loaded microemulsion.

As shown in Figure 4 and Table 1, a high local drug concentration was achieved in a short time, and the drug concentration-time profile showed little fluctuation; the ratio of the highest concentration (C_max_) to the lowest concentration was less than 2. This observation indicates that the drug molecules were released in a sustained manner from the microemulsion and permeated into the skin, which contributed to the long plateau period of drug concentration. Similarly, local drug concentration fluctuations in the microemulsion combined with microneedles group were small. Notably, by virtue of the microneedles used to enhance permeation, the local concentration of the drug in vivo increased sharply in the microemulsion combined with microneedles group, and the C_max_ and AUC_0–t_ of aconitine were approximately 3 times that of the microemulsion group. After a 10-h dialysis, no obvious inflammation occurred in the microemulsion-treated skin and the tissue surrounding the microdialysis probe (Figure 5). This suggests that the tested microemulsion had good biocompatibility with the skin and that microdialysis did not cause significant damage to the area. However, the drug was not detected in the samples obtained from the aconitine aqueous solution, indicating that the skin permeation of aconitine was greatly improved by using the microemulsion as a transdermal vehicle.

### 3.4. Intracellular Co-localization

**Figure 6:**
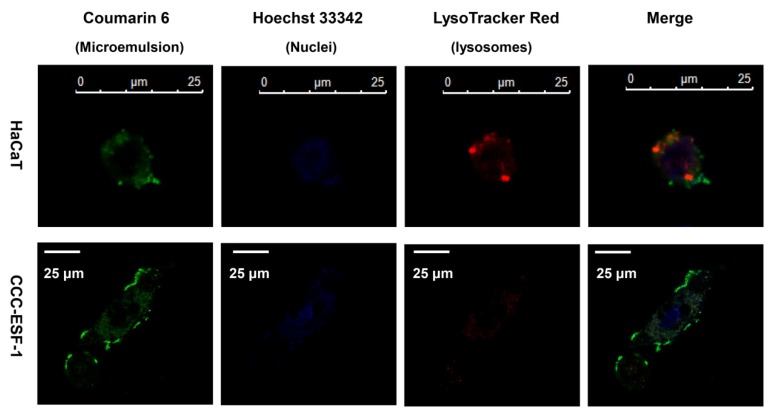
Intracellular localization of coumarin 6-loaded microemulsion in HaCaT and CCC-ESF-1 cells; microemulsion, nuclei, and lysosomes were labeled with coumarin 6 (green), Hoechst 33342 (blue), and LysoTracker Red (red); the yellow shows green (microemulsion) colocalized with LysoTracker Red.

The HaCaT cells were smaller than the CCC-ESF-1 cells, with less cytoplasm and a larger nucleus-to-cytoplasm ratio. After incubation with the coumarin 6-labeled microemulsion, the HaCaT and CCC-ESF-1 cells exhibited similar uptake behavior from the microemulsion (Figure 6). The green fluorescence of coumarin 6 was mainly distributed in the cell membrane and cytoplasm and did not enter the nucleus. The red fluorescence of the lysosomal marker (LysoTracker Red) overlapped with the green fluorescence of the coumarin 6 in the cells, which appeared yellow under the laser confocal microscope, suggesting that the microemulsion was internalized by the cells and released the fluorescent substances in the cytoplasm via the lysosomes.

### 3.5. Cell Viability

**Figure 7:**
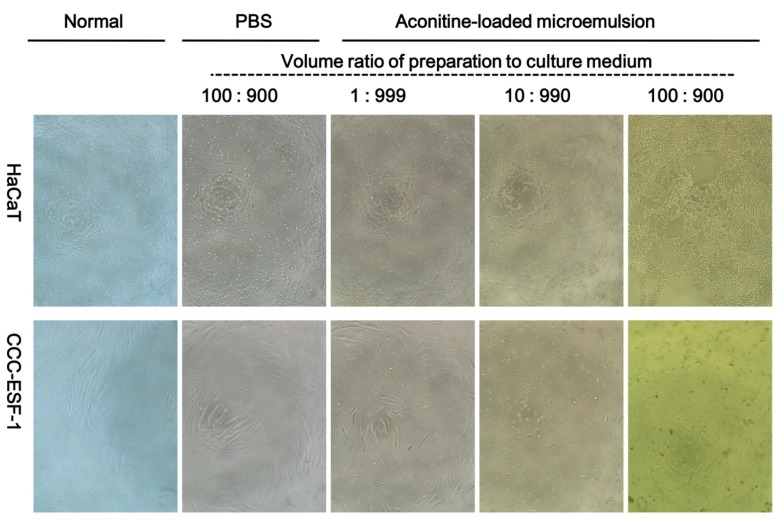
HaCaT and CCC-ESF-1 cell morphology after being incubated with aconitine-loaded microemulsion for 24 h. Magnification ×100.

In vitro release tests showed that aconitine in the microemulsion was completely released within 24 h, in addition, transdermal microemulsion is usually administered once or several times a day, and then the administration region will be washed, so only the cytotoxicity for 24 h was evaluated, which is close to the microemulsion that skin cells may contact during normal administration time. Normal HaCaT cells are epithelial-like and CCC-ESF-1 cells are spindle-shaped or irregular triangles. Both cells were full-shaped, adherent, and proliferated rapidly. After incubation with the aconitine-loaded microemulsion, the cells shrunk and rounded, and the number of pseudopods decreased. With increased concentration of the preparations, the cell shrinkage became more extensive, the cell number decreased, and some dead cells were suspended in the medium (Figure 7). With the same high concentration of preparation, the necrosis of the CCC-ESF-1 cells was more severe than that of the HaCaT cells.

**Figure 8:**
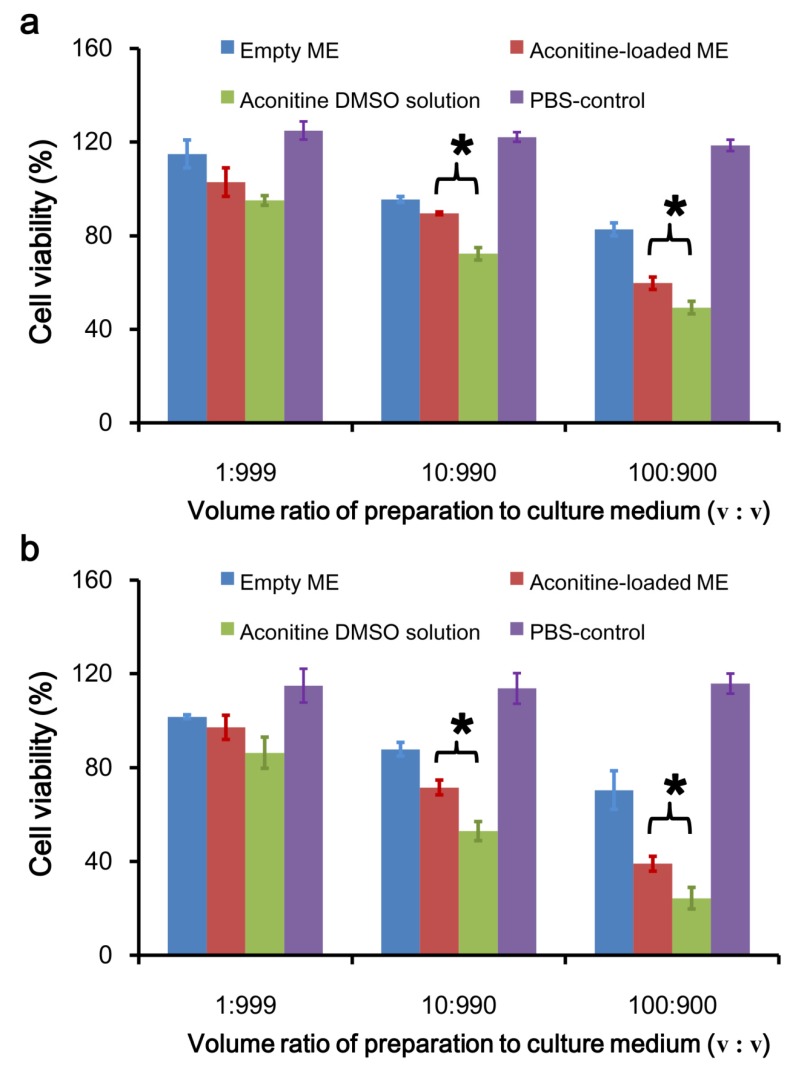
HaCaT (**a**) and CCC-ESF-1 (**b**) cell viability after being treated with various preparations for 24 h (ME, microemulsion; PBS-control, PBS without aconitine; * *p* < 0.05; *n* = 3).

The cell viability results are consistent with the cell morphology observations (Figure 8). In order to evaluate the possible effect on the cells of the dilution of the dispersion medium (PBS) in the microemulsion, an equal volume of PBS as a microemulsion was used as a control group in the experiment. The results showed that the added amount of PBS had no significant effect on cell morphology and growth. At low concentrations, microemulsions without aconitine had slight effects on cell viability, and there was no significant difference compared with the control group (*p* > 0.05), but as the concentration increased, cell viability gradually decreased. Aconitine exhibited a certain degree of toxicity to the cells. Therefore, after aconitine was loaded, the microemulsion toxicity increased. The cytotoxicity of each aconitine preparation was also concentration-dependent. Compared with the aconitine DMSO solution group, the aconitine microemulsion group produced less cytotoxicity (*p* < 0.05). In addition, at the same preparation concentration, the CCC-ESF-1 cells exhibited lower viability than the HaCaT cells.

### 3.6. Skin Irritation

**Figure 9:**
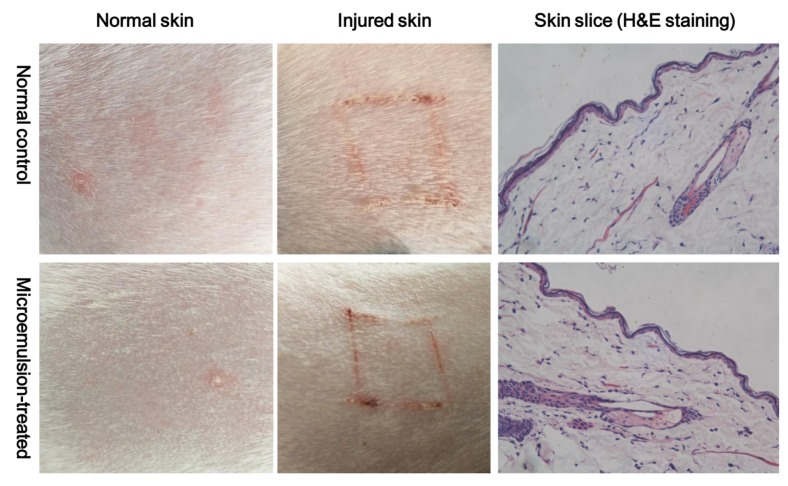
Normal and injured rat skin after being treated with aconitine-loaded microemulsion in vivo once a day for 7 days (H&E, hematoxylinandeosin). Magnification ×400.

After 7 consecutive days of transdermal administration in vivo, normal skin and damaged skin showed no obvious irritant reaction, and the microstructure of the skin showed that the cell morphology of each layer was normal and no inflammatory reaction had occurred (Figure 9).

## 4. Discussion

In the present work, microemulsion combined with microneedles was employed for enhancing transdermal drug delivery, and in vivo microdialysis was used to assess the permeability of the aconitine-loaded microemulsion for topical administration. Since aconitine is a liposoluble drug, an oil-in-water microemulsion was prepared to increase its solubility. 

A previous study revealed that microemulsion droplets are disorganized during permeation into the stratum corneum, which may result in quick drug release and generate a relatively high local drug concentration in 30 min [19]. Drug molecules released from a microemulsion solution with low viscosity permeate into the skin mainly by passive diffusion [20]. Furthermore, drug transdermal delivery should be consistent with Fick’s first law of diffusion (J = KDC_0_/h, where, J is the transdermal flux, D is the drug diffusion coefficient, K is the partition coefficient, and C_0_ is the drug concentration in the preparation) [21]. In addition, the microemulsion can disrupt the “brick wall” structure of the stratum corneum, which would both increase the distribution and diffusion coefficients of aconitine and enhance transdermal drug delivery [22,23]. Several studies have confirmed that drug-loaded nanoparticles can release drugs after entering the hair follicle [24,25]. The hair follicle has a three-dimensional structure and a large internal surface area, which can increase the percutaneous absorption of a drug. This may be an important way for the nanoparticles to improve the percutaneous absorption of a drug, which provides a new mechanism for transdermal delivery of microemulsion-enhancing drugs [26]. A relatively constant transdermal flux was generated, and time, as well as the drug diffusion coefficient, had little effect on the subcutaneous local drug concentration. However, the drug concentration in the microemulsion and the drug distribution coefficient can greatly affect the local drug concentration.

Assisted by microneedles, the transdermal drug amount and the transdermal drug flux are both significantly increased, indicating excellent enhanced permeation. The results of the current in vivo microdialysis study are consistent with the above reasoning since the aconitine concentration in the local tissue achieved a steady state within a short time that was maintained over a long period. This observation suggests that the drug diffused through the skin while it was transported to the tissue with a similar flux.

By loading into the microemulsion, the biosafety of aconitine toward skin cells is improved, which may be due to the sustained-release of the drug from the nanocarriers, further reducing the cell’s uptake of aconitine, which has been demonstrated in our previous work [27]. The relatively constant subcutaneous aconitine distribution obtained by using the microemulsion as a nanocarrier for transdermal delivery may be conducive to diminishing the toxicity induced by the drastic fluctuation of drug concentration in the body.

Aconitine has strong side effects and a narrow treatment window [28]. During transdermal administration using conventional preparations, due to poor drug permeability, the dose is often increased to achieve therapeutic effects, which is likely to cause adverse reactions. In this current work, although the use of microemulsions increased the transdermal permeation of drugs, it was still difficult to precisely control the amount of transdermal drug delivery. The combination of microneedles and microemulsions for the transdermal administration of aconitine can accurately control the dosage, thereby avoiding serious, toxic side effects, and effectively improving the safety of the medication. Besides, the microneedles used in our study have been widely used in skin care, and a number of metal microneedle products have been approved by the FDA for use in adjuvant injections [29,30]. Therefore, this technique has value as a potential clinical application.

## 5. Conclusions

In combination with microneedle administration, microemulsions can be directly introduced into the skin. During the process of passing through the skin, encapsulation of the drug in microemulsions can reduce drug uptake into skin cells, thereby reducing skin irritation and increasing local delivery of drugs to enhance local therapeutic effects. In addition, this study confirms that in vivo local microdialysis suitable for evaluating nanocarriers including microemulsions and microneedle-assisted transdermal administration.

## Figures and Tables

**Table 1:** Pharmacokinetic parameters of aconitine loaded into microemulsion sampled using microdialysis after application on rat abdominal skin in vivo (*n* = 3).

Parameter	Unit	Microemulsion	Microemulsion-Microneedles
C_max_	μg/mL	1.51 ± 0.03	4.04 ± 0.12 **
AUC_0–t_	min/μg/mL	684.76 ± 19.01	2026.62 ± 10.20 **
MRT_0–t_	min	303.07 ± 27.32	311.46 ± 2.04

Abbreviations: T_max_, time to peak concentration; C_max_, peak concentration; AUC, area under the concentration-time curve; MRT, mean residence time. Compared with microemulsion group, ** *p* < 0.01.
